# Diagnostic Challenges and Management of Relapsing Polychondritis with Large-Airway Involvement: A Case Series and Literature Review

**DOI:** 10.3390/life14091194

**Published:** 2024-09-21

**Authors:** I-Chun Kuo, Chen-I Hsieh, Yi-Chan Lee, Li-Jen Hsin, Wan-Ni Lin, Michael J. Rutter

**Affiliations:** 1 Department of Otolaryngology-Head & Neck Surgery, Chang Gung Memorial Hospital, Taoyuan 333, Taiwan; ickuo@cgmh.org.tw (I.-C.K.); lijen.hsin@gmail.com (L.-J.H.); y1829@cgmh.org.tw (W.-N.L.); 2 College of Medicine, Chang Gung University, Taoyuan 333, Taiwan; b9002063@cgmh.org.tw; 3 Division of Rheumatology, Allergy and Immunology, Chang Gung Memorial Hospital, Taoyuan 333, Taiwan; dr.rheum.jen@gmail.com; 4 Department of Otolaryngology-Head & Neck Surgery, Chang Gung Memorial Hospital, Keelung 204, Taiwan; 5 Division of Pediatric Otolaryngology, Aerodigestive and Esophageal Center, Cincinnati Children’s Hospital Medical Center, Cincinnati, OH 45229, USA

**Keywords:** relapsing polychondritis, airway, subglottic stenosis, microlaryngobronchoscopy, tracheostomy

## Abstract

Objectives: Our aim was to investigate the diagnostic challenges and management of relapsing polychondritis (RP) with airway involvement, highlighting the need for accurate diagnosis and effective intervention to prevent severe complications. Methods: In this retrospective study, medical records from January 2011 through June 2024 at a single tertiary-care institution were reviewed. This study was approved by the institutional review board. A total of 34 patients were diagnosed with RP, among whom 4 presented with significant airway complications. This study focused on these four patients, detailing their clinical presentations, diagnostic processes, and outcomes following various interventions. Results: All patients were initially misdiagnosed with asthma and later developed severe airway issues necessitating interventions such as tracheotomy and endotracheal intubation. Diagnostic imaging, microlaryngoscopy and bronchoscopy (MLB) were crucial for identifying subglottic stenosis and other airway alterations. Treatments included high-dose steroids, rituximab, and surgical interventions such as balloon dilation and tracheostomy. Only one patient could be decannulated; the other three remained dependent on tracheostomy and experienced significant complications due to emergency medical interventions. Conclusions: RP can manifest with nonspecific respiratory symptoms similar to asthma, which may delay correct diagnosis and appropriate treatment, leading to critical airway complications. The early, precise identification of RP, particularly with airway involvement, is vital. MLB and dynamic expiratory CT scans play significant roles in clinical diagnosis and management. A multidisciplinary approach involving otolaryngologists, rheumatologists, and pulmonologists is essential for optimizing patient outcomes and minimizing complications.

## 1. Introduction

Relapsing polychondritis (RP) is a rare systemic disorder predominantly affecting cartilaginous structures and proteoglycan-rich tissues, such as the auricles, nasal cartilage, ribs, larynx, trachea, and joints. This immune-mediated disease is characterized by recurrent episodes of inflammation, leading to episodic pain, progressive anatomical deformities, and functional impairments. The auricle is the most frequently affected site and is involved in approximately 90% of patients, whereas the nasal cartilage is the second most common site of involvement, with an initial diagnostic incidence of 24% [1,2,3].

The challenge in diagnosing RP lies in its nonspecific clinical manifestations and the absence of specific laboratory tests, histologic patterns, or imaging studies that definitively indicate ongoing inflammation. Consequently, the average diagnostic delay of approximately 2.9 years contributes to irreversible tissue damage and adversely impacts prognosis [4]. This report describes four patients who were initially diagnosed with asthma due to persistent coughing but subsequently developed acute airway compromise, requiring emergent interventions such as tracheotomy and endotracheal intubation. The clinical course and presentation are meticulously documented to highlight diagnostic challenges and management strategies, providing essential insights into early warning signs that could help avert airway crises.

## 2. Materials and Methods

Medical records from January 2011 through June 2024 were reviewed, identifying 34 patients diagnosed with RP. Among them, four patients developed severe airway complications. Institutional review board approval was obtained for this study (IRB #202301789B0), with a waiver of informed consent. A comprehensive review of medical records was conducted to evaluate the disease course, clinical presentation, and findings from laryngoscopy and computed tomography (CT) imaging.

## 3. Results

### 3.1. Case 1

A 45-year-old woman who denied systemic disease had experienced intermittent, nonproductive coughing for one year and had received inhaled corticosteroids (ICSs) for asthma control. She sometimes also experienced voice changes and anterior neck soreness and once had shortness of breath after an upper respiratory infection (URI) and was admitted to the internal medicine ward. Intravenous steroid therapy initially alleviated her breathing difficulties, but her condition progressively worsened over the following days, with an eventual need for endotracheal intubation. Once extubation was attempted, severe respiratory distress recurred within hours postextubation, ultimately necessitating emergency tracheotomy. A head-and-neck CT scan was arranged under the impression of upper airway obstruction. CT scans revealed cricoid mucosal thickening with edematous changes. The patient subsequently underwent microlaryngoscopy and bronchoscopy (MLB), which revealed edematous vocal folds and a narrowed subglottis. Balloon dilation was introduced once to the subglottis; although the lumen easily dilated, it collapsed immediately after balloon catheter withdrawal. The patient’s subglottic mucosa was intact with no scarring but was severely swollen. She also complained of left sternal pain. A review of her medical history revealed that she had presented with a nontraumatic nasal-bone deformity several years prior, for which she underwent rhinoplasty at an outside hospital (OSH).

Since then, the patient had been tracheostomy-tube-dependent and had received oral steroid treatment for suspected RP with airway involvement.

Three years after disease diagnosis, newly emerging midtracheal stenosis occurred after the patient was changed to a cuffed tracheostomy tube for general anesthesia for spine surgery after a car accident. A Dumon stent was applied after multiple failed attempts at balloon dilatation for tracheal stenosis. The patient was dependent on a tracheostomy tube and had a tracheal stent in place. Due to persistent left sternal pain and bilateral hip soreness, a bone scan was arranged and revealed bilateral sacroiliitis with left sternoclavicular joint arthritis. Sacroiliitis is particularly unusual in the context of RP. However, conditions such as rheumatoid arthritis, ankylosing spondylitis, and psoriatic arthritis are known to present with sacroiliitis. Rheumatology specialists re-evaluated these conditions, and there is currently no definitive evidence supporting a diagnosis of any of these diseases. It is possible that her sacroiliitis is related to post-traumatic changes following a recent car accident.

### 3.2. Case 2

A female patient presented at 18 years of age with hoarseness, chronic cough, and intermittent shortness of breath for six months. She had been initially diagnosed with asthma and treated with ICS, but the condition persisted, leading to multiple hospitalizations for intravenous pulse steroid therapy. She had also experienced ear pain and hearing impairment, at which time otitis externa was diagnosed, but no significant improvement was observed with antibiotic treatment. A review of her history of four hospitalizations revealed that the patient had undergone two endotracheal tube intubations for acute exacerbation of dyspnea and extubation after steroid therapy. The fourth admission culminated in acute respiratory distress and required an emergency tracheotomy to maintain the airway due to difficult transoral intubation, which was complicated by pneumothorax and postobstructive pulmonary edema. Several months later, the patient developed a noticeable nasal deformity and complained of bilateral knee pain. Three years later, she underwent surgery for bilateral cataracts. She currently retains a tracheostomy tube for airway support. A small larynx disproportional to the patient’s age was observed following a nasopharyngoscopy examination in the outpatient department.

She was initially treated with oral prednisolone, followed by a transition to biologic drugs. The first biologic, a TNF inhibitor (Humira), provided suboptimal efficacy. The patient was then switched to abatacept, which resulted in several months of stable disease control. However, a subsequent worsening of nasal and auricular symptoms prompted the initiation of rituximab therapy. Following this adjustment, the patient’s condition stabilized, and rituximab was maintained as the ongoing treatment.

A URI occurring the previous year was complicated with right middle lobe pneumonia, and unexpected tracheal rupture manifested at the emergency department while the patient was being changed to a cuffed tracheal tube for ventilator support. Subcutaneous emphysema, pneumomediastinum, and pneumothorax deteriorated her oxygen saturation, and extracorporeal membrane oxygenation (ECMO) was emergently applied for life support. A Dumon Y-stent was subsequently applied for tracheal repair but was complicated by intractable bronchial granulation. The patient currently relies on a tracheostomy tube and has a stent installed for the trachea and main bronchus.

### 3.3. Case 3

An 18-year-old man experienced a nonproductive cough combined with globus and hoarseness for six months. He had also smoked for approximately one year. He initially visited a pulmonologist and an otorhinolaryngologist and was diagnosed with asthma and smoking-related vocal edema.

The patient was once admitted to the ward for pneumothorax after vigorous coughing and was subsequently discharged with ICS for asthma control. Intractable cough and dyspnea periodically challenged him for several months, and he was hospitalized for several rounds of intravenous methylprednisolone. The cough and dyspnea were alleviated immediately after steroid therapy but repeatedly occurred after discharge. The patient also experienced painful swelling in his left ear and eventually underwent surgical debridement for a suspected auricle infection. However, the swollen auricle appeared deformed after surgery, and the swelling did not resolve.

During the last admission, sudden airway collapse occurred after an uncontrollable cough; emergency tracheotomy was performed to secure the airway but was complicated by tracheal and esophageal lacerations. A CT scan revealed thickening of and enhancement in the cricoid-thyroid cartilage with resultant airway stenosis, indicating an inflammatory process. Esophageal repair and MLB were performed, revealing a small larynx disproportionate to the patient’s age and severe edematous vocal folds. The narrowed subglottic lumen was dilated by the airway balloon but collapsed immediately after the balloon catheter was retrieved. No granulation or scar tissue was observed in the subglottis, but a swollen and pale luminal mucosa was observed. The patient is now dependent on a tracheal tube for airway support and treatment with oral prednisone and immunosuppressants.

### 3.4. Case 4

A 51-year-old woman had experienced intermittent shortness of breath and dyspnea on exertion for 5 years. She underwent a cardiac and airway evaluation and was diagnosed with subglottic stenosis (SGS). Airway balloon dilation was first applied, but her breathing difficulties persisted. She progressed to severe dyspnea two weeks after receiving a second course of airway balloon dilation. Elective tracheostomy was executed to secure the airway. Laser laryngomicrosurgery (LMS) and balloon dilation were applied for SGS at the same time. She recovered 1 month later in a tolerable respiratory condition. The patient had had a notably deformed nose since childhood but did not pay much attention to it. At clinic visits during the following 9-year period, she complained of intermittent, painful bilateral ankle swelling and persistent but tolerable breathing difficulties. Progressive conductive hearing impairment was also found. A few months prior to this visit, her dyspnea worsened substantially after a URI attack, and CT of her airway with three-dimensional reconstruction revealed circumferential soft tissue thickening of the subglottis, resulting in concentric SGS. Revised elective tracheostomy was suggested, but the patient was lost to follow-up because she was reluctant to undergo tracheostomy.

## 4. Discussion

Diagnosing RP is complex due to its diverse clinical manifestations and the absence of a definitive diagnostic test [5]. The diagnosis is typically established through a combination of clinical evaluation and the exclusion of other conditions with similar presentations. Referral to a rheumatologist or specialist in autoimmune diseases is advisable to confirm the diagnosis and develop an effective treatment plan. The primary clinical differential diagnoses for RP include granulomatosis with polyangiitis (GPA) and newly identified autoinflammatory diseases, such as vacuoles, E1 enzyme, X-linked, autoinflammatory, somatic (VEXAS) syndrome, which have been discovered through advances in pangenomic sequencing. Both GPA and VEXAS syndrome share features of inflammatory diseases characterized by chondritis or cartilage destruction. Although several diagnostic criteria sets, such as McAdam’s [3], Damiani and Levine’s [6], and Michet’s [1] criteria, have been suggested, they are limited by the lack of formal validation, small sample sizes, and vague definitions of organ involvement. Therefore, a thorough diagnostic approach that integrates clinical findings, laboratory tests, and imaging studies is crucial in identifying RP. Given the diagnostic complexities and challenges, a more in-depth comparative study is being conducted to analyze and compare the manifestations of airway involvement in various autoimmune diseases.

In managing RP, a comprehensive battery of laboratory tests is pivotal for diagnosis and surveillance. A complete blood count (CBC) is fundamental for detecting anemia and leukocytosis, which are indicators of inflammation in RP. Moreover, elevated C-reactive protein (CRP) levels and an increased erythrocyte sedimentation rate (ESR) are nonspecific markers employed to evaluate the intensity of inflammation and track disease progression. Despite the absence of RP-specific autoantibodies, screenings for antinuclear antibodies (ANAs) and antineutrophil cytoplasmic antibodies (ANCAs) are instrumental in excluding other autoimmune conditions. Additionally, renal function tests and urinalysis are crucial for identifying kidney involvement. Although infrequent, assessment of complement levels (C3, C4) can provide insights into the activation of the complement system in some patients. Given the lack of specific autoimmunity-related markers closely associated with RP, these laboratory evaluations are often utilized to assess the presence of concomitant diseases and monitor disease activity [7,8] (Table 1).

Cartilage biopsy is generally not necessary for diagnosing relapsing polychondritis but can be useful in differential diagnosis when other conditions are suspected. Histological findings typically include inflammatory infiltration in the perichondrium and cartilage matrix, loss of normal cartilage architecture, chondrocyte disruption, fibrosis, and granulation tissue formation. These features vary with disease stage and severity. However, biopsy alone is insufficient for diagnosis, necessitating clinical correlation and integration with other diagnostic criteria. While of limited routine value, cartilage biopsy may be indicated in cases with unilateral auricular symptoms, lack of systemic involvement, or poor response to glucocorticoids to exclude other diagnoses [7,9].

Although airway cartilage involvement is initially less common, occurring in approximately 10% of cases, approximately 50% of patients may develop airway involvement as the disease progresses [4]. According to a previous study, positive findings from chest CT and bronchoscopy revealed that airway involvement in these patients was more than 80% [10]. The cardiovascular and respiratory complications arising from RP are linked to significant morbidity and mortality [11]. While the availability of interventional treatments has contributed to improved prognosis, RP-related morbidity and mortality remain significant, especially in cases with tracheobronchial disease. Recent studies showed that these patients required more intensive treatment, faced higher infection risks, and were often admitted to intensive care [12].

The respiratory manifestations of RP may initially present as chronic cough, hoarseness, or alterations in voice quality, accompanied by vocal fold edema [13]. These symptoms may progress to dyspnea and stridor and are responsive to steroids. High doses of steroids can alleviate these symptoms, but discontinuation of the medication often leads to their recurrent exacerbation. These respiratory signs may or may not be associated with nasal or auricular cartilage deformities, representing the pleomorphic and variable presentations at different stages of the disease, thus posing a diagnostic challenge owing to the pleomorphic nature and insidious onset of the disease. Furthermore, because of their responsiveness to steroids, these conditions can be easily misdiagnosed as asthma, potentially leading to delayed treatment or acute respiratory distress [10,14].

Bronchoscopy is accompanied by an increased risk of exacerbation of airway inflammation and may induce potentially fatal respiratory distress; consequently, its use is limited to selected cases [15]. According to Lee et al. [16], dynamic expiratory CT should be considered a routine diagnostic component if there is clinical suspicion of airway involvement. Dynamic expiratory CT scans are utilized to identify tracheal and main bronchomalacia, defined as a reduction in the cross-sectional area exceeding 50%, and air trapping, indicated by an insufficient increase in lung parenchyma attenuation during expiration. Inspiratory CT scans are assessed for tracheal and bronchial stenosis, characterized by more than 25% luminal diameter narrowing compared with unaffected segments, as well as for wall thickening greater than 2 mm and calcification.

**Figure 1:**
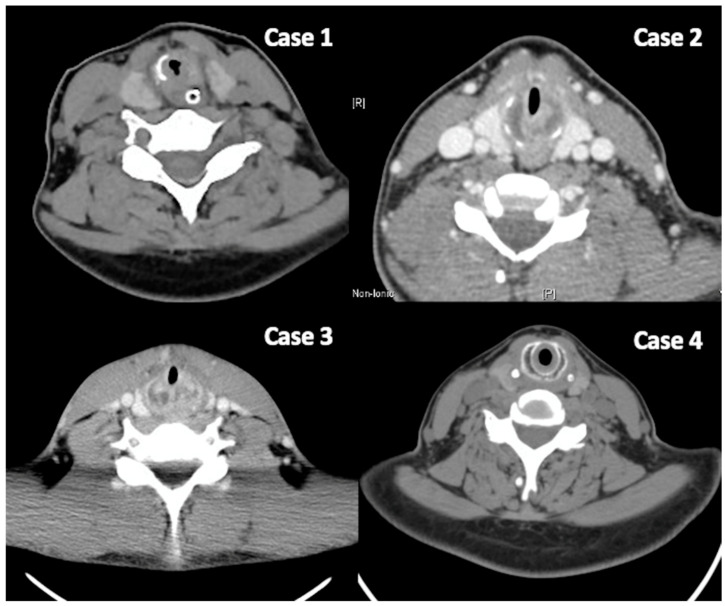
CT images revealed a thickened and edematous subglottic mucosa, along with destruction and deformation of the cartilage, indicating disease-induced damage.

In the subglottic area, which is the only part of the respiratory tract comprising a complete ring of cartilage (cricoid cartilage), swelling or narrowing often causes relatively severe respiratory distress and may necessitate tracheostomy to maintain airway patency. In cases of RP with subglottic involvement, CT images can reveal thickening and edema of the subglottic mucosa and even destruction and deformation of the cartilage itself, suggesting disease-induced damage (Figure 1). In the larynx, in addition to mucosal edema, there is also visible destruction and deformation of the arytenoid cartilage. Differentiating between airway involvement due to GPA and relapsing polychondritis can be particularly challenging. Research indicates that GPA-related stenoses are typically subglottic and circumferential, and tend to occur in the subglottic region, whereas stenoses associated with relapsing polychondritis are more likely to be tracheal, anterior, calcified, and often extend to the bronchi, as seen on chest CT [17].

In addition to CT images, MLB performed to evaluate the airway is a more appropriate approach for diagnosis. MLB is conducted in the operating room with spontaneous or controlled patient breathing. When a clinical suspicion of laryngeal and airway lesions exists, presenting as atypical or inexplicable severe asthma accompanied by hoarseness or unexplained laryngeal/vocal cord edema, MLB allows for a comprehensive assessment of the airway by direct visualization and palpation, as well as measurement of the airway size [18]. This facilitates the determination of the nature and extent of the pathological findings and airway obstruction, enabling decision making regarding the possible removal of intubation, the maintenance of a breathing tube, or even the planning of an elective tracheostomy, thereby effectively reducing the incidence of emergency tracheostomy following premature extubation.

Magnetic resonance imaging (MRI) is a valuable tool for assessing the extent of tracheal and laryngeal involvement, as it can differentiate chronic fibrosis and inflammation from acute edema and inflammation. MRI is considered more sensitive than CT in distinguishing fibrosis, which appears as low signal intensity on both T1- and T2-weighted images, from inflammation, which shows high signal intensity on T2-weighted images and enhancement after gadolinium administration. However, the utility of MRI is limited by its lower spatial resolution compared to that of 5 CT for detailed evaluation of the airway wall [19,20].

Fluorodeoxyglucose (FDG) positron emission tomography (PET)/CT scans can detect active cartilaginous lesions at any stage of relapsing polychondritis, although their low spatial resolution is a limitation. PET/CT can also uncover unsuspected cartilaginous involvement. While the full evaluation of PET/CT’s efficacy is ongoing, monitoring the standard uptake value (SUV) may help distinguish between active and fibrotic lesions, particularly in patients with isolated tracheobronchial involvement. Additionally, 18F-fluorodeoxyglucose PET/CT can be useful for assessing the extent of the disease, including asymptomatic cartilaginous involvement. However, its role in monitoring the therapeutic response in relapsing polychondritis remains controversial [21,22,23].

**Figure 2:**
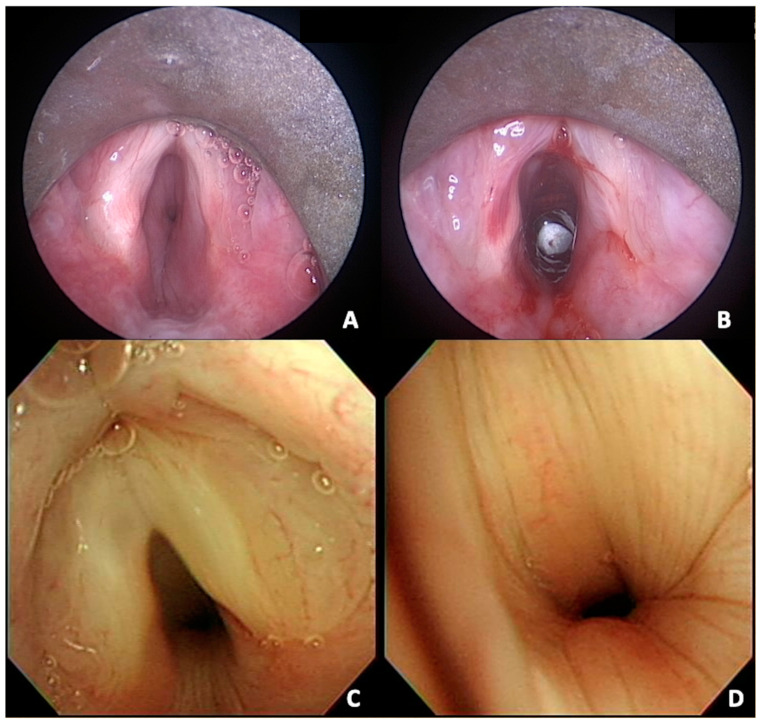
MLB in Patient 1 demonstrated laryngeal edema and significant subglottic narrowing (**A**). After balloon dilation, the subglottis showed no obvious scarring, with subglottic collapse indicating a lack of cartilaginous support (**B**). Patient 3 also exhibited similar edema (**C**) with no evident scar formation (**D**).

All four patients underwent emergency tracheotomy/elective tracheostomy, and three of them subsequently underwent MLB for a comprehensive evaluation of the respiratory tract. Only one patient was decannulated successfully, albeit with mild and tolerable breathing difficulties. Intraoperatively, vocal fold edema and subglottic channel swelling with narrowing were observed, differing from typical cicatricial stenosis. The subglottic mucosa was edematous but intact, without evident scar formation (Figure 2). Endoscopically guided balloon dilation was successfully performed to expand the airway; however, significant recollapse and luminal narrowing occurred once the balloon catheter was removed. Therefore, the acute-phase presentation of the subglottis is likely due to inflammation of the cricoid cartilage, causing loss of structural support and collapse.

The presentation of RP with airway involvement may manifest as stenosis or malacia, requiring careful interventional treatment to avoid iatrogenic trauma. For severe airway stenosis and malacia, airway stenting may be considered. However, long-term follow-up has shown that stent-related complications are almost inevitable. These complications include stent fracture, stent migration, pulmonary artery or airway erosion, bleeding, and granulation tissue ingrowth [24]. Additionally, airway malacia may extend from the central to the peripheral airways, necessitating the implantation of multiple stents. Handa et al. reported that the survival rate in patients who received stenting was significantly lower than that in the non-stenting group, with a notably lower mortality rate observed in the non-stenting group [11]. The long-term use of metallic stents significantly increases the risk of fatal complications. Consequently, the U.S. Food and Drug Administration (FDA) recommends silicone stents over metallic stents for benign central airway obstruction [25]. In light of these risks, careful assessment is essential before deciding to place a stent. When stenting is deemed necessary, silicone stents are preferred for long-term outcomes.

For cicatricial SGS, grade I–II stenosis is treated endoscopically, including techniques such as laser and balloon dilation. However, for more severe cases (grades III–IV), open reconstructive surgery is recommended [26]. This type of surgery commonly involves the use of rib cartilage as a graft. However, in patients with RP, it is crucial to consider that repeated inflammation may have compromised the structural integrity of the cricoid and rib cartilage, causing loss of the supportive cartilaginous scaffold. Consequently, traditional reconstructive approaches with laryngotracheoplasty (LTP) may not yield satisfactory outcomes. Maintaining a tracheostomy or placing a T tube may be preferable strategies before disease stabilization.

In some patients, once airway stability is achieved, decannulation of the tracheostomy tube may be considered. Nevertheless, after more than ten years of clinical follow-up, there remains a risk of progression to respiratory failure necessitating tracheostomy. When the trachea and bronchi are also affected and damaged, leading to obstruction and narrowing, endobronchial stenting may also be necessary [27].

In addition to disease-related recurrent inflammation leading to cartilaginous structure degradation and further damage to the respiratory tract, patients with chronic inflammation and instability of the respiratory structures are susceptible to severe complications due to medical interventions. For example, in Patient 1, tracheostomy tube replacement led to midtracheal stenosis; Patient 2 experienced tracheal rupture necessitating emergency use of ECMO and tracheal stenting; and Patient 3 had severe tracheal rupture during emergency airway establishment, extending to esophageal injury. These experiences underscore the need for cautious assessment and management of such patients in clinical settings. It is advisable that procedures be conducted by otolaryngologists to minimize the risk of severe complications.

This case report on RP patients with large-airway involvement has significant limitations because of its retrospective nature and the small sample size of only four patients, which may restrict the generalizability of the findings. These constraints underscore the need for larger, prospective studies to validate these findings and ensure the development of management strategies that are effective and applicable to a wider spectrum of patients with RP.

## 5. Conclusions

This case series highlights the critical nature of airway involvement in RP, often misdiagnosed as asthma, requiring urgent interventions like tracheotomy. It emphasizes the need to consider RP in differential diagnoses for patients with atypical respiratory symptoms resistant to standard asthma treatments.

MLB and dynamic expiratory CT are essential for assessing airway integrity and guiding timely management decisions. Effective RP management involves controlling inflammation with glucocorticoids, conventional disease-modifying antirheumatic drugs (DMARDs) and biologic drugs, along with proactive airway monitoring [28]. Understanding the immunological mechanisms behind airway inflammation and stenosis is crucial for developing targeted therapies.

Standardized treatment protocols and comprehensive evaluations of airway interventions, including tracheostomy and bronchial stenting, are needed to improve patient outcomes. Preventing complications from airway procedures requires ongoing research. A multidisciplinary approach involving otolaryngologists, rheumatologists, and pulmonologists is vital for optimal care and improving patient quality of life.

## Figures and Tables

**Table 1:** Characteristics, symptoms, and laboratory data of patients with relapsing polychondritis and large-airway involvement.

Characteristics	Case 1	Case 2	Case 3	Case 4
Sex	F	F	M	F
First affected site	Nose	Larynx	Larynx	Nose
Affected site				
Oculus		v		v
Auricle		v	v	
Inner ear (hearing)		v		v
Nose	v	v		v
Larynx/trachea	v	v	v	v
Bronchus	v	v	v	v
Joint	v	v		v
Emergent tracheotomy	v	v	v	
Complications from airway intervention	Midtracheal stenosis	Pneumothorax/pulmonary edema; tracheal rupture	Tracheal and esophageal laceration	
Time from respiratory symptoms to respiratory failure	1 year	4 months	6 months	5 years
Laboratory data at the time of airway intervention				
CBC (M: 3.9–10.6; F: 3.5–11 1000/µL)	7.1	20.6 H	15.9 H	8.0
CRP (<5 mg/L)	9.1 H	7.89 H	121.65 H	<5
ESR (0–30 mm/hr)	11	99 H	34 H	33 H
ANA (≤1:80)	Negative	Negative	Negative	Negative
ANCA (negative)	Negative	Negative	Negative	+(P-ANCA) H
Creatine (M: 0.64–1.27; F: 0.44–1.03 mg/dL)	0.47	0.49	0.94	0.68
C3 (90–180 mg/dL)	141	128	105	131
C4 (10–40 mg/dL)	37.6	29.7	23.9	30.5

Notes: This table summarizes the demographic, clinical, and laboratory characteristics of four patients presenting with relapsing polychondritis with large-airway involvement. Each column represents a distinct case, labeled Case 1 through Case 4. CBC, complete blood count; CRP, C-reactive protein; ESR, erythrocyte sedimentation rate; ANA, antinuclear antibodies; ANCA, antineutrophil cytoplasmic antibodies; C3, complement component 3; C4, complement component 4.

## Data Availability

The data presented in this study are available on request from the corresponding author.

## References

[1] Michet C.J., McKenna C.H., Luthra H.S., O’Fallon W.M. (1986). Relapsing polychondritis. Survival and predictive role of early disease manifestations. Ann. Intern. Med..

[2] Isaak B.L., Liesegang T.J., Michet C.J. (1986). Ocular and systemic findings in relapsing polychondritis. Ophthalmology.

[3] McAdam L.P., A O’Hanlan M., Bluestone R., Pearson C.M. (1976). Relapsing polychondritis: Prospective study of 23 patients and a review of the literature. Medicine.

[4] Trentham D.E., Le C. (1998). Relapsing polychondritis. Ann. Intern. Med..

[5] Zhang L., Wu T.-G., He Y.-J., Guo J.-Y., Han L.-S., Lu J.-M., Liu S.-Y., Li T.-F. (2020). Diagnosing relapsing polychondritis remains a common challenge: Experience from a Chinese retrospective cohort. Clin. Rheumatol..

[6] Damiani J.M., Levine H.L. (1979). Relapsing polychondritis—Report of ten cases. Laryngoscope.

[7] Mertz P., Costedoat-Chalumeau N., Ferrada M.A., Moulis G., Mekinian A., Grayson P.C., Arnaud L. (2024). Relapsing polychondritis: Clinical updates and new differential diagnoses. Nat. Rev. Rheumatol..

[8] Rednic S., Damian L., Talarico R., Scirè C.A., Tobias A., Costedoat-Chalumeau N., Launay D., Mathian A., Matthews L., Ponte C. (2018). Relapsing polychondritis: State of the art on clinical practice guidelines. RMD Open..

[9] Dawudi Y., Benali K., Beydon M., Hourseau M., Sacre K., Papo T. (2022). B-cell lymphoma mimicking relapsing polychondritis. Br. J. Haematol..

[10] Zhai S., Guo R., Zhang C.-M., Yin H., Wang B., Wen S. (2023). Clinical analysis of relapsing polychondritis with airway involvement. J. Laryngol. Otol..

[11] Handa H., Ooka S., Shimizu J., Suzuki N., Mineshita M. (2023). Evaluation of airway involvement and treatment in patients with relapsing polychondritis. Sci. Rep..

[12] Dion J., Costedoat-Chalumeau N., Sène D., Cohen-Bittan J., Leroux G., Dion C., Francès C., Piette J. (2016). Relapsing Polychondritis Can Be Characterized by Three Different Clinical Phenotypes: Analysis of a Recent Series of 142 Patients. Arthritis Rheumatol..

[13] de Montmollin N., Dusser D., Lorut C., Dion J., Costedoat-Chalumeau N., Mouthon L., Chassagnon G., Revel M.-P., Puéchal X. (2019). Tracheobronchial involvement of relapsing polychondritis. Autoimmun. Rev..

[14] Hazra N., Dregan A., Charlton J., Gulliford M.C., D’cruz D.P. (2015). Incidence and mortality of relapsing polychondritis in the UK: A population-based cohort study. Rheumatology.

[15] Ernst A., Rafeq S., Boiselle P., Sung A., Reddy C., Michaud G., Majid A., Herth F.J., Trentham D. (2009). Relapsing polychondritis and airway involvement. Chest.

[16] Lee K.S., Ernst A., Trentham D.E., Lunn W., Feller-Kopman D.J., Boiselle P.M. (2006). Relapsing polychondritis: Prevalence of expiratory CT airway abnormalities. Radiology.

[17] Jalaber C., Puéchal X., Saab I., Canniff E., Terrier B., Mouthon L., Cabanne E., Mghaieth S., Revel M.-P., Chassagnon G. (2022). Differentiating tracheobronchial involvement in granulomatosis with polyangiitis and relapsing polychondritis on chest CT: A cohort study. Arthritis Res. Ther..

[18] Myer C.M., O’Connor D.M., Cotton R.T. (1994). Proposed grading system for subglottic stenosis based on endotracheal tube sizes. Ann. Otol. Rhinol. Laryngol..

[19] Sato R., Takahashi H., Terasaki M., Okamoto S., Terasaki T., Toko H., Yagishita M., Hagiawara S., Kondo Y., Tsuboi H. (2021). Advantage of Magnetic Resonance Imaging in Detecting Tracheal Involvement and Evaluation of the Therapeutic Response in Relapsing Polychondritis With Asthma-Like Symptoms. J. Clin. Rheumatol..

[20] Thaiss W.M., Nikolaou K., Spengler W., Spira D., Xenitidis T., Henes J., Horger M. (2016). Imaging diagnosis in relapsing polychondritis and correlation with clinical and serological data. Skeletal Radiol..

[21] Sharma A., Kumar R., Mb A., Naidu G.S.R.S.N.K., Sharma V., Sood A., Dhir V., Verma R., Singh H., Bhattacharya A. (2020). Fluorodeoxyglucose positron emission tomography/computed tomography in the diagnosis, assessment of disease activity and therapeutic response in relapsing polychondritis. Rheumatology.

[22] Okuda S., Hirooka Y., Itami T., Nozaki Y., Sugiyama M., Kinoshita K., Funauchi M., Matsumura I. (2021). FDG-PET/CT and Auricular Cartilage Biopsy Are Useful for Diagnosing with Relapsing Polychondritis in Patients without Auricular Symptoms. Life.

[23] Zeng Y., Li M., Chen S., Lin L., Li S., He J., Wang J. (2019). Is 18F-FDG PET/CT useful for diagnosing relapsing polychondritis with airway involvement and monitoring response to steroid-based therapy?. Arthritis Res. Ther..

[24] Wu X., Zhang X., Zhang W., Huang H., Li Q. (2019). Long-Term Outcome of Metallic Stenting for Central Airway Involvement in Relapsing Polychondritis. Ann. Thorac. Surg..

[25] U.S. Food and Drug Administration (2005). FDA Public Health Notification: Complications from Metallic Tracheal Stents in Patients with Benign Airway Disorders. https://stening.es/download/articles/FDA-complication-from-metallic-tracheal-stents.pdf.

[26] Jefferson N.D., Cohen A.P., Rutter M.J. (2016). Subglottic stenosis. Semin. Pediatr. Surg..

[27] Hong G., Kim H. (2013). Clinical characteristics and treatment outcomes of patients with relapsing polychondritis with airway involvement. Clin. Rheumatol..

[28] Petitdemange A., Sztejkowski C., Damian L., Martin T., Mouthon L., Amoura Z., Cutolo M., Burmester G.R., Fonseca J.E., Rednic S. (2022). Treatment of relapsing polychondritis: A systematic review. Clin. Exp. Rheumatol..

